# Association Between First Episode Schizophrenia, Metabolic Syndrome and Insulin Resistance-Related Proteins in Female Balb/C Mice

**DOI:** 10.22086/gmj.v0i0.692

**Published:** 2018-04-01

**Authors:** Haseeb Sattar, Huqun Li, Yong Han, Hong Zhou, Sanaz Darbalaei, Weiyong Li

**Affiliations:** ^1^Department of Clinical Pharmacy, Wuhan Union Hospital Affiliated to Tongji Medical College, Huazhong University of Science and Technology, Wuhan, China

**Keywords:** Insulin Resistance, Impaired Glucose Tolerance, Metabolic Syndrome, Schizophrenia

## Abstract

**Background:**

Metabolic syndrome is a group of different disorders mainly includes, insulin resistance, obesity, cerebrovascular disorders, dyslipidemia, which leads to increase mortality. Patients suffering from related psychotic disorders such as schizophrenia are at the higher risk of developing metabolic syndrome. The aim of this study was to evaluate the association between the first episode of schizophrenia, metabolic syndrome and insulin resistance-related proteins in blood and adipose tissue of mice.

**Materials and Methods:**

Twelve, female Balb/c mice were randomly divided into two groups; one group was injected intraperitoneal MK-801(0.6mg/kg/d) to induce schizophrenia, and other group received the 0.9% normal saline for two weeks. Body weight, fasting blood glucose (FBG), oral glucose tolerance (OGT), and Homeostatic model assessment (HOMA), were observed. Blood and adipose tissue were collected and Western blotting was done to evaluate the insulin resistance related proteins (GGPPS, FAT, PTP-1B, GRK2, ATGL, FGF21, and PGC-1α) by using GAPDH as an internal standard.

**Results:**

There was a significant increase in mean body weight in schizophrenic group (21.76 vs 22.81, P=004). On day 14, the FBG, insulin concentrations and Homeostatic model assessment and insulin resistance (HOME-IR) were high in schizhphrenic group vs control group, e.g. 5.3±0.6 vs 3.47±0.2 (P=0.0001), 28.9±2.2 vs 23.3±0.6 (P<0.005) and 9.2±1.3 vs 3.9±0.2 (P=0.0001) . Impaired glucose tolerance deranged from 4.8mmol/L to 6.4mmol/L. Western blotting showed a marked increase in the expression of GGPPS, FAT, ATGL, and FGF21 proteins in monocytes and PTP-1B, GRK2, and PGC-1α ratios in adipose tissues.

**Conclusion:**

There was a positive relation between schizophrenia and metabolic syndrome e.g. insulin resistance and obesity. Certain proteins in adipocytes and blood were responsible for causing insulin resistance.

## Introduction

The prevalence of metabolic syndrome among schizophrenic patients is poorly understood. Dietary and lifestyle modifications, hypercortisolemia, alter lipid and carbohydrate metabolism are the few explanations for the development of metabolic syndrome [1].

One of the main consequence of metabolic syndrome is insulin resistance [2].

Recently, some discovered proteins such as geranylgeranyl diphosphate synthase (GGPPS), fatty acid translocase (FAT), protein tyrosine phosphatase-1B (PTP-1B), G-protein–coupled receptor kinase-2 (GRK2), adipose triglyceride lipase (ATGL), fibroblast growth factor 21 (FGF21) and peroxisome proliferator-activated receptor gamma coactivator 1-alpha (PGC-1α) in mice adipocytes and muscle tissues that were found to be associated with the pathogenesis of insulin resistance.

Abnormal PGC-1 α activity is likely to play a major role in the pathogenesis of hyperglycemia, insulin resistance, and cardiomyopathy. Increasing activity of PCG-1α causes insulin resistance in an animal model [3]. Previous animal studies revealed that expression of GGPPS expression was found to be higher in insulin-resistant adipose tissues, which activate MAPK/Erk1/2 pathway through Ras-prenylation leads to insulin resistance [4]. The ATGL is mostly present in adipose tissue, where it regulates lipolysis. Inhibition of ATGL in mice adipocytes may result in impaired fatty acids release from adipocytes but improves glucose and insulin hemostasis [5]. The FAT/CD36 regulates long chain fatty acids in cardiac and skeletal muscles; it promotes insulin sensitivity [6]. The PTP1B has a negative role in insulin signaling pathways, and its increased intracellular expression lead to insulin resistance in an animal model [7]. The GRK2 is also involved in inhibition of insulin signaling pathways, which results in insulin resistance [8]. Most of the studies have been conducted on animal models to evaluate the relationship between insulin resistance and insulin resistance related proteins, but very few studies are available in which schizophrenic animal model is used. The GGPPS, FAT, PTP-1B, GRK2, ATGL, FGF21 and PGC-1α proteins have been studied for the pathogenesis of insulin resistance by evaluating these proteins either in muscle or fat tissues, but no study have been performed to evaluate these protein levels in blood monocytes.This study was designed to assess the relationship between the first episode of schizophrenia and metabolic syndrome, and whether after the development of metabolic syndrome, these proteins levels have any impact on insulin resistance in the schizophrenic animal model and finally to show the relationship between the expression of these proteins in adipocytes and monocytes.

## Materials and Methods

### Animal Sample and Study Design

Twelve 8-weeks-old female inbred Balb/c strain mice (6 mice in each group), weighing 20-22g were purchased from Beijing, China Fukang Biotechnology Co., Ltd. Animals were randomly divided into two groups. One group was treated with once daily intraperitoneal injection of 0.6mg/kg MK801 (dizocilpine maleate, Sigma, USA) to induce schizophrenia, while the other group was treated with intraperitoneal 0.9% normal saline as a control group. Each group was treated for two weeks and was housed in a cage separately in a specific pathogen-free environment with controlled humidity (50±10%), room temperature (22±1°C) and illumination for 12 hrs. The experiment was carried out according to the European Community Guidelines for the Use of Experimental Animals and received approval from HUST Animal Experiment Ethical Committee.

### Spontaneous Activity and Stereotypic Behavior

Homemade open field test box (100cm × 100cm × 50cm) was used to determine spontaneous activity and to establish schizophrenia, stereotypic behavior e.g. licking, biting, head movement and smelling activities were also observed as previously described [9].

### Determine Fasting Blood Glucose (FBG) and Insulin Level

The FBG level was checked using a Glucometer (Accu-Chek®, USA), Insulin concentrations were detected by using Mouse Insulin ELISA assay kit (Sibayagi, Gunma, Japan) according to manufacturer guidelines.

### Measurement oral Glucose Tolerance Test (GTT) and Insulin Resistance

ice were fasted for 12hrs after which 2g/kg glucose solution was given orally to each group.

Blood glucose concentrations were noted after, 15min, 30min, 60min, 90min, and 120min by using a Glucometer (Accu-Chek®, USA). Homeostatic model assessment (HOMA-IR) values for each mice were calculated by using the following formula: HOMA-IR = (fasting glucose × fasting insulin)/ 22.5)[10].

### Blood and Tissue Sample Collection

Mice were killed by giving an intraperitoneal injection of 200 mg/kg sodium pentobarbital. Blood samples were collected from orbital sinus of each mice in eppendorf (EP) tube by removing eyeball. Subcutaneous white and brown fat tissues were harvested and stored in -80°C refrigerator.

### Western Blotting

Seven different type of insulin resistance related proteins e.g. GGPPS, FAT, PTP-1B, GRK2, ATGL, FGF21, and PGC-1α were detected (GADPH as an internal standard) and analyzed by western blotting. Briefly, adipose tissues were rinsed 3-4 times with phosphate buffer solution to remove blood. The RIPA buffer (10 µl/1ml) containing protease inhibitors (phosphatase inhibitor , US ASPEN Ace model number AS1004), and PMSF Cell Signaling (US ASPEN Ace model AS1006) at 4°C (100 nM), were used to extract proteins from adipose and plasma (Monocytes). Rabbit GGPPS, FAT, PTP-1B, GRK2, ATGL, FGF21, and PGC-1α antibodies (ABCAM, China) were used as primary antibodies (1:1000,1:2000, 1:1000, 1:1000, 1:2000, 1:500 and 1:2000) and HRP-Goat anti-Rabbit antibody (KPL, SeraCare, Beijing China) at 1:10000 was used as secondary antibody. For optical density value, AlphaEaseFC software was used.

### Statistical Analyzed

Data was presented as mean ± standard deviation (SD) by using SPSS 19.0

statistical software. Data were analyzed by Two-sample t-test and ANOVA. The P<0.05 was considered statistically significant.

## Results

Spontaneous activity and stereotypic behavior After two-weeks administration of MK-801, schizophrenic group showed increased spontaneous activity 23.25±5.27 vs 62.28±3.36 (Control vs MK-801) (P=0.0001) and also demonstrated more stereotypic behavior than control e.g 0.3±0.26 vs 2.8±0.1 (P=0.0001) respectively. (Tables-1 and 2).

**Table-1:** Spontaneous Activity Observed in Mice Model

**Groups**		**P-value**
**Control**	**schizophrenic**	
19.91±14.75	61.53±25.03	0.032
18.55±16.32	58.00±24.21	0.001
24.27±17.21	63.73±31.15	0.001
30.27±25.10	65.87±19.99	0.001

**Table-2:** Stereotypic Behavior Control V.S Schizophrenic Group

**Groups**		**P-value**
**Control**	**schizophrenic**	
0.5±0.527	2.7±0.483	<0.05
0.0±0.000	2.8±0.422	<0.05
0.4±0.211	2.9±0.316	<0.05

### Mean Body Weight

Weight gain is one of the risk factor for the development of metabolic syndrome. Two weeks after the treatment, mean body weight of schizophrenic group was found to be increased (22.81g ±1.05) as compared to the control group (21.76g ±0.45) which was statistically significant (P=0.004).

### The FBG and Insulin Levels

In schizophrenic group, high serum fasting glucose levels (5.36±0.67mmol/L) were observed (P<0.001, 95% CI -2.5260 to -1.2540) as compared with the control (3.47±0.25mmol/L) group.

**Figure-1:**
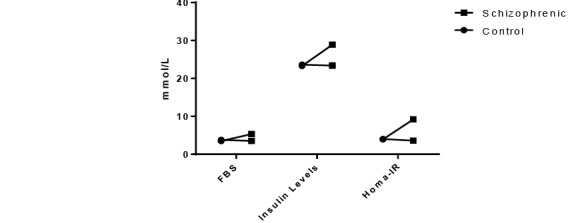
FBS(Fasting Blood Glucose), Insulin and Homa-IR Levels at 0 and Day 14

In the schizophrenic group, the mean serum insulin concentrations were higher than of the control group (28.9±0.72mmol/L vs. 23.3±0.68mmol/L), which were statistically significant (P<0.005). The HOMA-IR model demonstrated that schizophrenic group had greater insulin resistance (9.2±1.3mmol/L,) as compared to the control group (3.9±0.2mmol/L CI: 5.7919-7.0081) (Figure-1).

### The Oral GTT

**Figure-2:**
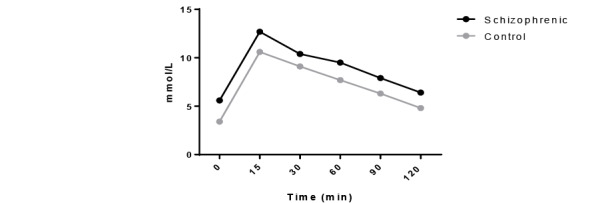
Oral Glucose Tolerance Test, after 2 hours of oral glucose intake

After 120mins of oral glucose ingestion, impaired glucose tolerance was observed in schizophrenic group (6.4mmol/L) (Figure-2).

### Western Blotting

**Figure-3:**
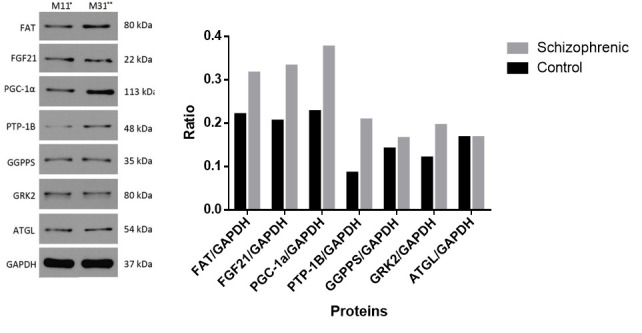
Expression of seven different proteins in Adipose Tissue. GAPDH was used as internal standard. *Control group, **Schizophrenic group

**Figure-4:**
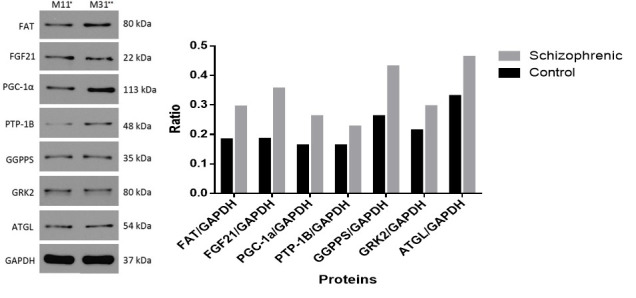
Expression of seven different proteins in Blood (Monocytes). GAPDH was used as internal standard. *Control group, **Schizophrenic group

Western blotting was performed on both blood samples and adipose tissue to detected GGPPS, FAT, PTP-1B, GRK2, ATGL, FGF21, and PGC-1α proteins. The relative content ratio of proteins were calculated. The GGPPS expression was high in the blood (P<0.05). In adipose tissue, PTP -1B expression was relatively higher than other proteins. PGC-1α and GRK2 expression were low in blood sample while high in adipose tissue (P<0.05). While FGF21 expression was high in blood but low in the adipose tissue. The FAT and ATGL expression in adipose tissue and blood samples differed significantly (P<0.05) (Figures-3 and 4).

### Ratio of Relative Proteins

**Figure-5:**
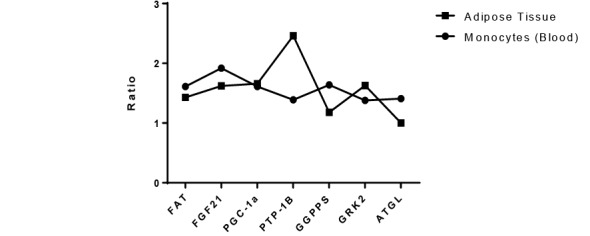
Proteins ratio comparison between blood(monocytes) and adipose tissue. (protein ratio in Schizophrenic/ protein ratio in Control)

Relative proteins ratio was calculated by the following equation, ratio of protein in schizophrenic group/ratio of protein in the control group. In the blood, GGPPS, FAT, FGF21 and ATGL proteins ratios were found to be high as compared to adipose tissue while PTP-1B, PGC-1α, and GRK2 were significantly high in adipose tissue (Figure-5).

## Discussion

Our study demonstrated that the psychotic illness such as schizophrenia was highly associated with the prevalence of metabolic syndrome e.g. obesity and insulin resistance. Earlier research has descriptively described the association between psychotic disorders and metabolic abnormalities and cardiovascular disorders, presenting that people with the psychotic disease might be more prone to disorders such as dyslipidemia, obesity, and glucose intolerance e.g. diabetes, with a higher incidence of metabolic syndrome comorbidities [11]. The prevalence of the metabolic syndrome among the subjects of normal weight was noted as 60%, while among obese and overweight were 60% and 22%, respectively [12]. According to recent study the incidence of metabolic syndrome among first episode schizophrenia was 13% while it is more common in elderly patients (39.2%, CI = 32.6%–46.1%) [17]. another study demonstrated the similar results that the patients with mental illness have higher rate of development of metabolic syndrome (32.6%) [18].

our study also revealed the similar results as the mice with induced schizophrenia exhibit higher rate of metabolic syndrome than control e.g. increased body weight (P=0.004) and increased insulin resistance(P=0.001).

In our study, we found that certain proteins levels were higher in the schizophrenic group than control group, which suggests their role in the pathogenesis of insulin resistance e.g. GRK2 protein level was found to be high in adipose tissue. Previous studies showed that increased GRK2 levels were found in mononuclear cells in patients with metabolic syndrome while Tamoxifen triggered GRK2 deletion in mice leads to reversal of insulin resistance, improve fasting and impaired glucose tolerance [13]. The GGPPS involved in the mevalonate pathway, promotes free fatty acids induced insulin resistance in skeletal muscle and decreasing GGPPS improves insulin resistance [14]. The FAT/CD36 is responsible for long chain fatty acids (LCFA) uptake in skeletal and heart muscles and higher LCFA uptake rate has been detected in skeletal muscle of type 2 diabetic and obese patients. Different studies suggested that impaired FAT regulation can lead to triacylglycerol accumulation and insulin resistance [6]. The ATGL is present abundantly in adipocytes and macrophages. Schoiswohl *et al*. conducted a study on ATGL knockout mice. They found that reduced ATGL expression led to improved FBG, oral GTT, greater hepatic insulin signaling and decreased insulin resistance [15].

The PTP-1B is an intracellular phosphatase, and its overexpression can lead to insulin resistance. In our study, PTP-1B ratio was high in both blood and adipose tissue in schizophrenic group. Recently a study was conducted to investigate the effects of PTP-1B antagonist Fumosorinone, isolated from *Isaria fumosorosea*. This study revealed that inhibition of PTP-1B leads to decrease serum glucose, lipid levels and decrease insulin resistance in mice [7].

The most interesting results which we found were FGF21 ratios, which was high in the schizophrenic group as compared to the control animals. Previous studies suggested that FGF21 has synergistic effects with insulin. It promotes insulin sensitivity and may cause weight loss [16].

In contrast, our results showed the increased level of FGF21 in adipose tissue of schizophrenic group might be due to some compensatory effects which further need to be evaluated.

## Conclusion

Finally, our study suggests that there was a positive relationship between the first episode of schizophrenia and metabolic syndrome. Impaired proteins ratio e.g. GGPPS, FAT, PTP-1B, GRK2, ATGL, FGF21 and PGC-1α presented in adipocytes and blood is responsible for causing insulin resistance and weight gain. For the future perspective, detecting serum levels of these proteins in schizophrenic patients might be used as a tool to establish insulin resistance.

## Conflict of interest

Authors declared no conflict of interest.
